# Parent-adolescent informant discrepancy on the Strengths and Difficulties Questionnaire in the UK Millennium Cohort Study

**DOI:** 10.1186/s13034-023-00605-y

**Published:** 2023-05-11

**Authors:** Charlotte Booth, Dario Moreno-Agostino, Emla Fitzsimons

**Affiliations:** 1 Centre for Longitudinal Studies, University College London, London, UK; 2 ESRC Centre for Society and Mental Health, King’s College London, London, UK

**Keywords:** Informant discrepancy, Mental health, Adolescence, Strengths and Difficulties Questionnaire, Latent difference score

## Abstract

**Background:**

Developmental researchers often use a multi-informant approach to measure adolescent behaviour and adjustment, but informant discrepancies are common. In general population samples, it is often found that parents report more positive and less negative outcomes than adolescents themselves. This study aimed to investigate factors associated with informant discrepancy, including adolescent sex, and parental level of psychological distress and education.

**Methods:**

Informant discrepancy on the Strengths and Difficulties Questionnaire was investigated using a Latent Difference Score (LDS) approach, which estimates the true difference between parent and adolescent reports in a structural equation model. The sample were parent-adolescent dyads from the seventh wave of the UK Millennium Cohort Study (*N* = 6947, 49.3% female, aged 17 years).

**Results:**

Parents reported lower levels of difficulties (emotion symptoms, peer problems, conduct problems), and higher levels of pro-social behaviour than adolescents themselves. Conditional effects were found, as discrepancy was greater amongst parent-daughter dyads for emotion and peer problems, and greater amongst parent-son dyads for conduct problems and pro-social behaviour. Parent-adolescent discrepancy was also greater generally if parents had a lower level of psychological distress or a higher level of education.

**Conclusions:**

In a large general population sample from the UK, it was found that adolescents tended to report more negative and less positive outcomes than parents reported about them. Conditional effects were found at the parent and adolescent level suggesting that specific informant biases are likely to impact the measurement of adolescent behaviour and adjustment across reporters.

**Supplementary Information:**

The online version contains supplementary material available at 10.1186/s13034-023-00605-y.

Developmental researchers are generally in agreement about the benefits of using a multi-informant approach, such as from parents, teachers, and self-report, to measure child and adolescent adjustment, which can provide better precision than single reports alone [1, 18, 49]. However, studies consistently report large discrepancies and only low to moderate levels of agreement between informants, resulting in problems of interpretation and leading researchers to question the measurement properties of certain instruments [2, 35].

The Strengths and Difficulties Questionnaire (SDQ) is a widely used, relatively short, multi-informant measure of child and adolescent adjustment, capturing five domains including emotion symptoms, peer problems, conduct problems, hyperactivity-inattention, and pro-social behaviour, in youth aged 3–17 years [16]. A recent meta-analysis found cross-informant agreement on the SDQ to be in the low to moderate range in general population samples between teacher–child reports (*r* = 0.19–0.33), parent-teacher reports (*r* = 0.26–0.46), and parent–child reports (*r* = 0.30–0.38) [2]. Concordance rates on the SDQ have been shown to be higher than other multi-informant measures, such as the Child Behaviour Checklist [17], and tend to exceed levels considered acceptable for cross-informant agreement [2], however large informant discrepancies persist.

Cross-informant agreement on the SDQ tends to be highest for externalising difficulties, such as hyperactivity-inattention, and lowest for internalising difficulties, such as emotion symptoms [2]. This finding is often attributed to the fact that externalising difficulties are more easily observed, compared to internalising difficulties, which may be hidden or unobserved [10, 44]. However, this does not always apply, as a wide array of factors can explain differences in rates of informant discrepancies across domains. For example, conduct problems are considered more observable, yet may be underreported due to social desirability [23], or non-disclosure of certain behaviours to parents, such as antisocial or illicit adolescent behaviour [5]. And, in some cases, internalising problems may be well reported by parents, such as when the parent–child relationship is strong, characterised by more time spent together, including open communication [47].

Studies in clinical samples, of children presenting to services with psychopathology, tend to find higher levels of cross-informant agreement between parents and children, than those found in general population samples [2]. The direction of informant discrepancies also differs between clinical and general population samples, as parents tend to report higher symptoms than children in clinical samples [7, 20], whereas children tend to report higher symptoms than parents in general population samples [12, 24, 44]. This suggests that parents of children in clinical settings may be more aware of their children’s difficulties, which could be attributed to the fact that parents usually facilitate access to clinical services [25].

Reasons for informant discrepancies are likely to be wide ranging, and have been summarised in theoretical models, such as the Attribution Bias Context (ABC) model [10]. This model posits that informant discrepancies may arise due to (a) differences in attribution of the cause of children’s behaviour (e.g., situational vs dispositional), (b) individual informants’ own cognitive biases (e.g., depression-distortion hypothesis), and (c) the reporting context of the informant (e.g., at school or at home) [10]. The latter has often been used to describe parent-teacher discrepancies, as informants report from either the home or the school context, providing valuable information across settings [13].

Mental health, in particular that of mothers, has been identified as a potential source of informant discrepancy. Early research in this area, which found that mothers with depression reported higher symptoms in their children than mothers with no depression, concluded that mothers with depression tended to overreport their children’s symptoms due to their own mental health problems [4, 39]. However, these reports were strongly criticised for lack of empirical evidence and misinterpretation of results [40]. Firstly, higher mental health difficulties would be expected in households where either the parent or child suffers with mental health difficulties, due to bi-directional effects and heightened psychological distress [28]. Secondly, parents with a history of mental health difficulties may be more aware of their children’s distress and sensitive to signs of maladjustment, resulting in more accurate reporting of children’s symptoms compared to parents with no history of mental health difficulties [8].

In summary, while parent reports of adolescent mental health and adjustment are widely used, they are not directly comparable to adolescent self-report. Both types of report are prone to error due to subjective bias and differences in reporting context, and neither can be considered as the gold standard [10]. It is important to understand the factors associated with informant discrepancies, in order to inform models of reporter bias and improve the interpretation of adolescent adjustment measures.

## The current study

In the current study, parent-adolescent informant discrepancy was investigated using data from the seventh wave of the Millennium Cohort Study (MCS), collected in 2018–2019 when cohort members were aged 17, reflecting the only timepoint when both parent and self-reported SDQ were collected. In line with previous research, it was hypothesised that parents would report lower difficulties and higher pro-social behaviour than adolescents [12, 24]. Conditional effects were investigated, and it was hypothesised that discrepancy would be greater in parent-daughter dyads, due to the large increase in self-reported difficulties observed in older adolescent girls [36, 37, 42]. In addition, it was expected that informant discrepancies would be lower if parents reported a higher level of psychological distress themselves, due to increased understanding of mental health difficulties [8]. Parent’s highest level of education was also explored as a potential conditioning factor, as research tends to find that higher levels of parental education are protective for children’s mental health [43, 46], although to our knowledge this has not been explored as a moderator of discrepancy.

Latent Difference Score (LDS) modelling was used, which is considered a flexible and robust way to investigate informant discrepancies [9]. LDS models are a specific type of structural equation model, which infer unobserved (latent) constructs from observed (manifest) variables [27]. A true difference score can be estimated from the self and parent reported latent factors, which is considered free from measurement error [14]. The mean difference score, interindividual differences in change, and the covariance between the self-report and the difference score can then be included as model parameters for further investigation [14]. In addition, due to the multiple manifest variables observed within a latent model, measurement invariance across reporters can be tested and accounted for, to ensure that any differences observed are the result of a true difference and not variation in how parents and adolescents interpret and respond to items in a questionnaire [21].

## Method

### Participants and design

MCS data are freely available and were downloaded from the UK Data Service website. The sample used for the current study was smaller than the total number of participants at wave 7 (*N* = 10,757). Participants were 6947 parent-adolescent dyads who had at least partial SDQ data and matched demographic information at wave 7. Missing data was observed due to either failure to complete and return questionnaires (14.4%), or in some cases failure to match completed questionnaires to respective parent information (18.2%). To account for missing data, we created an Inverse Probability Weight (IPW), as the propensity to have complete data based on the observed characteristics of the child. The MCS data team already provide survey weights to account for non-response and attrition over time [11]. Therefore, we created a product of the new IPW and the existing MCS wave 7 survey weight, to account for both types of missing data. The sex distribution was equal among participants (49.3% female); although, parents were more likely to be female (85.6%). Most participants were ethnically White (86.2% vs 13.8% ethnic minority), which is representative of the population at baseline.

### Measures

The Strengths and Difficulties Questionnaire (SDQ) [19] was collected by self- and parent-report using the respective versions at wave 7, which have previously shown good psychometric properties [17]. The SDQ has five subscales in total, four measuring difficulties (conduct problems, hyperactivity-inattention, emotion symptoms, peer problems), and one subscale measuring pro-social behaviour. Each subscale contains five items, which are rated on a 3-point scale to indicate frequency (0 = ‘Never’, 1 = ‘Sometimes’, 2 = ‘Always’). Confirmatory Factor Analyses (CFA) were run to establish the most suitable factor structure for the data. The five-factor structure was found to be a poor fit for both the parent (CFI = 0.854, RMSEA = 0.049, SRMR = 0.047) and adolescent data (CFI = 0.831, RMSEA = 0.051, SRMR = 0.050). Therefore, univariate CFA were run for each subscale and reporter, which were found to be in the acceptable-good range for conduct problems, emotion symptoms, peer problems, and pro-social behaviour (Additional file 1: Table S1). Model fit was poor for hyperactivity-inattention across reporters and was not examined further, perhaps because this scale was tapping into multiple facets of impulsivity, which are not unidimensional. Internal consistencies are also reported in Additional file 1, which were all in the acceptable range.

The Kessler-6 [26] assessed parent’s level of psychological distress at wave 7. Parents were asked to indicate how often in the last 30 days they had been feeling (“down”, “depressed”, “hopeless”, etc.), using a 5-point scale (0 = ‘None of the time’, 1 = ‘A little of the time’, 2 = ‘Some of the time’, 3 = ‘Most of the time’, 4 = ‘All of the time’). The six items were summed to create a total score (range 0–24). Validated cut-off scores were applied to indicate greater than moderate (≥ 5) levels of psychological distress [26]. One third of parents exceeded the moderate level of distress (34.6%).

Parent’s highest level of education was collected at the baseline of the study. We used data collected at wave 2 (2004–2005), where a sampling boost was carried out, thus reflecting the first wave for some MCS families. If no information was available at wave 2, then information was augmented from wave 1. A binary variable was created to indicate whether parents had a university degree or equivalent vocational diploma (equal to a National Vocational Qualification (NVQ) Level 4 and above: 56.3%), versus a lower level of education (NVQ Level 3 and below: 43.7%).

### Statistics

Measurement invariance was tested initially using a series of nested multi-group confirmatory factor analyses [31]. Three models were compared: the baseline model tested for configural invariance (i.e., whether the same factor structure held across groups). If this level of invariance was found to hold based on the model fit [22], then metric invariance was tested, where item loadings were constrained to be equal across groups. Since LDS models concern comparisons of the factor means, scalar invariance (where loadings and intercepts of the items are constrained to be equal across groups) was deemed necessary to ensure comparisons to be free of measurement bias. The loss of fit after introducing any of these constraints was compared against usual criteria, i.e., loss in fit smaller than 0.010 and 0.015 in CFI and RMSEA, respectively [6].

If models were not deemed to hold based on the outlined criteria, a partial measurement invariance approach was used. This approach was implemented using modification indices, which inform about which parameters need to be freed to improve model fit. Since each of the latent variables under study was measured by five indicators, we allowed a maximum of two out of five of the measurement parameters to vary across groups, so the majority of items were invariant before establishing partial measurement non-invariance. Although there is no consensus on the thresholds for partial measurement invariance [38], ensuring invariance in the majority of indicators goes in line with recommendations by Vandenberg and Lance (48), and is slightly more conservative than other approaches where only two indicators are constrained to be invariant [45].

**Fig. 1:**
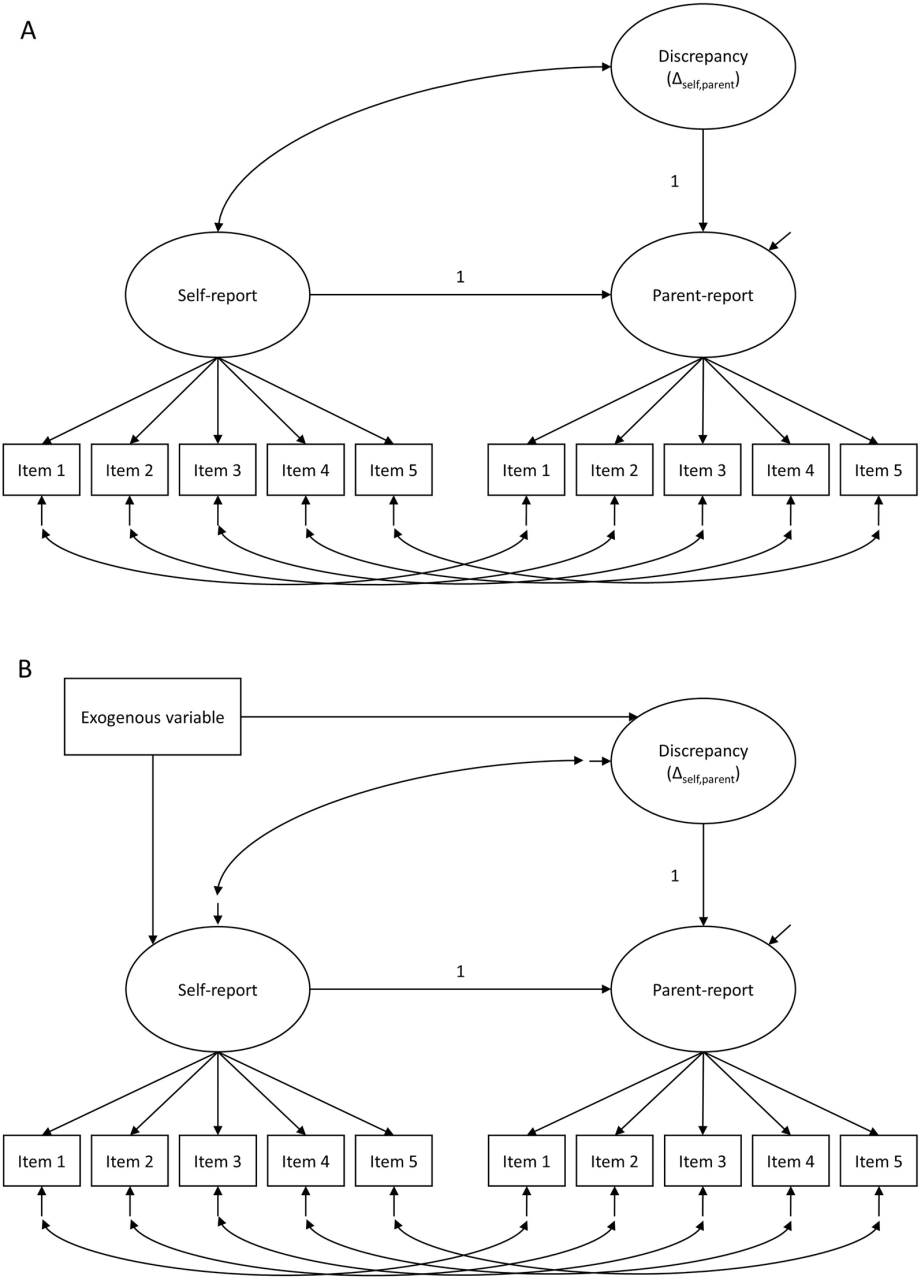
Graphical representation of unconditional (**A**) and conditional (**B**) LDS model

LDS models were then conducted on the best fitting model for each of the remaining constructs using the methods outlined in DeHaan et al. [9]. Using an LDS model specification, a latent factor for the self-report was measured by the self-reported indicators. Similarly, a parent-reported factor was measured with the parent-reported indicators, but the latent mean of that factor was constrained to be zero. By regressing this latent factor on the self-reported latent factor and adding a second-order latent variable measured by the parent-reported latent factor, this latter variable can be understood as the latent discrepancy (or difference) between the two informants’ latent factors. Positive scores on this factor represent higher parent-reported ratings and negative scores represent lower parent-reported ratings relative to self-report. A graphical representation of the unconditional LDS model is presented in Fig. 1A.

Conditional LDS models were then estimated to evaluate whether discrepancy varied as a function of adolescent sex (male/female), parental level of psychological distress (low/high), or parental level of education (low/high). To evaluate conditional effects, the latent self-report and the latent discrepancy factors were regressed on adolescent sex, parental psychological distress, and parental education, in separate models for each exogenous variable. This way, the relationship between the exogenous variables and the discrepancy factors (and, by extension, on the parent’s report) would be net of the potential differences on the self-report by those same exogenous variables. A graphical representation of the conditional LDS model is presented in Fig. 1B. The non-linear combinations of the conditional effects were obtained as appropriate for each of the levels of the exogenous variables, and the 95% confidence intervals of these conditional effects were plotted to visualise the direction of effects.

Analyses were conducted in R using lavaan version 0.6–12 [41]. In our main analyses, we used maximum likelihood (ML) estimation, which is compatible with applying survey weights. However, in order to account for item-level missingness and the non-normality of indicators, we conducted a separate set of analyses using MLR estimation and full information maximum likelihood (FIML) and report these in Additional file 2.

## Results

### Descriptive statistics

A descriptive comparison of the parent and adolescent version of the SDQ and item-level means, and standard deviations (SD) are presented in Table 1. In general, adolescent report showed higher item mean levels for difficulties and lower item mean levels for pro-social behaviour than parent report.

**Table 1:** Parent and adolescent version items, means, and standard deviations from the SDQ

Item	Parent version	Mean	SD	Adolescent version	Mean	SD
	Conduct problems	1.00	1.33		1.65	1.50
5	Often has temper tantrums or hot tempers	0.40	0.63	I get very angry	0.53	0.65
7	Generally obedient (r)	0.42	0.57	I usually do as I am told (r)	0.65	0.56
12	Often fights with other children	0.04	0.24	I fight a lot	0.15	0.40
18	Often lies or cheats	0.14	0.40	I am often accused of lying or cheating	0.19	0.46
22	Steals from home, school, or elsewhere	0.04	0.23	I take things that are not mine	0.11	0.35
	Hyperactivity-inattention	2.33	2.18		3.65	2.25
2	Restless, overactive	0.32	0.58	I am restless	0.90	0.69
10	Constantly fidgeting or squirming	0.23	0.52	I am constantly fidgeting	0.64	0.72
15	Easily distracted, concentration wanders	0.52	0.67	I am easily distracted	0.93	0.72
21	Thinks things out before acting (r)	0.65	0.62	I think before I do things (r)	0.68	0.58
25	Sees tasks through to the end (r)	0.57	0.64	I finish the work I am doing (r)	0.77	0.62
	Emotion symptoms	1.93	2.23		3.44	2.45
3	Often complains of headaches	0.39	0.63	I get a lot of headaches	0.45	0.65
8	Many worries	0.55	0.68	I worry a lot	1.07	0.77
13	Often unhappy, downhearted	0.27	0.55	I am often unhappy	0.47	0.65
16	Nervous or clingy in new situations	0.45	0.65	I am nervous in new situations	1.05	0.76
24	Many fears, easily scared	0.29	0.55	I have many fears	0.49	0.66
	Peer problems	1.67	1.75		2.10	1.69
6	Rather solitary, tends to play alone	0.50	0.67	I am usually on my own	0.58	0.68
11	Has at least one good friend (r)	0.16	0.45	I have one good friend or more (r)	0.16	0.42
14	Generally liked by other children (r)	0.20	0.44	Other people my age generally like me (r)	0.51	0.58
19	Picked on or bullied by other children	0.18	0.47	Other children or young people pick on me	0.13	0.38
23	Gets on better with adults than with other children	0.63	0.70	I get on better with adults than with people my age	0.70	0.69
	Pro-sociality	8.48	1.74		7.90	1.70
1	Considerate of other people’s feelings	1.71	0.48	I try to be nice to other people	1.79	0.42
4	Shares readily with other children	1.69	0.54	I usually share with others	1.49	0.59
9	Helpful if someone is hurt	1.78	0.46	I am helpful if someone is hurt	1.65	0.52
17	Kind to younger children	1.87	0.37	I am kind to younger children	1.77	0.46
20	Often volunteers to help others	1.44	0.64	I often volunteer to help others	1.19	0.64

Response categories (0 = Never, 1 = Sometimes, 2 = Often); r = reverse scored; Hyperactivity-inattention was not explored in the current study due to poor CFA model fit

### Measurement invariance

Measurement invariance was tested with a series of nested multi-group confirmatory factor analyses. Partial scalar invariance was established for all four factors, by using modification indices to identify the parameters that needed to be freed to improve model fit. Table 2 displays model fit indices and the specific items that were freed are presented in the note.

**Table 2:** Measurement invariance models between parent and self-report

	Fit			Fit difference		
	CFI	RMSEA	SRMR	ΔCFI	ΔRMSEA	ΔSRMR
Conduct						
Configural	0.934	0.038	0.030	–	–	–
Metric	0.901	0.044	0.043	0.032	0.006	0.014
Metric partial	0.932	0.037	0.032	0.002	0.001	0.003
Scalar	0.862	0.050	0.044	0.070	0.013	0.011
Scalar partial	**0.925**	**0.037**	**0.033**	**0.006**	**0.001**	**0.001**
Emotion						
Configural	0.968	0.048	0.028	–	–	–
Metric	0.967	0.046	0.030	0.001	0.002	0.002
Scalar	0.883	0.082	0.067	0.084	0.036	0.037
Scalar partial	**0.962**	**0.048**	**0.034**	**0.005**	**0.002**	**0.004**
Peer						
Configural	0.973	0.028	0.019	–	–	–
Metric	0.955	0.034	0.030	0.017	0.006	0.011
Scalar	0.766	0.074	0.058	0.189	0.040	0.028
Scalar partial	**0.949**	**0.036**	**0.031**	**0.007**	**0.001**	**0.001**
Pro-social						
Configural	0.984	0.027	0.018	–	–	–
Metric	0.969	0.035	0.031	0.015	0.008	0.014
Scalar	0.865	0.068	0.052	0.104	0.033	0.021
Scalar partial	**0.957**	**0.039**	**0.034**	**0.012**	**0.004**	**0.003**

Bold indicates selected model; Conduct problems: the loadings for item 12 (“often fights”) were freed from the metric model, the intercepts for item 5 (“temper/angry”) and item 18 (“lies/cheats”) were freed from the scalar model; Emotion symptoms: the intercepts for item 8 (“many worries”) and item 16 (“nervous/clingy”) were freed from the scalar model. Peer problems: the intercepts for item 14 (“generally liked”) and item 19 (“picked on”) were freed from the scalar model; Pro-social behaviour: the intercepts for item 1 (“considerate/nice”) were freed from the scalar model

### Latent difference scores

LDS models were run on the four partially invariant models (Table 3). Model fit was good in all cases. The latent difference scores were significant and negative for all difficulties (conduct, emotion, peer), showing that parents on average reported lower difficulties compared to adolescents, with the greatest discrepancy observed for conduct problems, and the smallest discrepancy observed for peer problems. The LDS for pro-social behaviour was significant and positive, showing that parents on average reported higher pro-social behaviour compared to adolescents. Variances were all significant, showing that there was significant heterogeneity in discrepancy scores. Covariances were all significant and negative, showing that in cases where the discrepancy factor was negative, the higher the intercept for self-reported difficulties, the greater discrepancy was observed, with the largest effect observed for emotion symptoms. However, for pro-social behaviour, the higher the intercept for self-report, the smaller the amount of discrepancy was observed.

**Table 3:** Latent difference score models between parent and self-report

	Model fit				Latent difference score		
	*N*	CFI	RMSEA	SRMR	Mean std	Variance	Covariance std
Conduct	6704	0.931	0.049	0.031	− 0.898***	0.097***	− 0.490***
Emotion	6690	0.956	0.061	0.039	− 0.424***	0.081***	− 0.579***
Peer	6697	0.946	0.044	0.030	− 0.195***	0.105***	− 0.349***
Pro-social	6757	0.949	0.050	0.038	0.506***	0.066***	− 0.477***

Maximum likelihood estimation with survey weights; Standardised means and covariances displayed for ease of interpretation; *p < .05, **p < .01, ***p < .001 level

### Conditional models

#### Adolescent sex

**Fig. 2:**
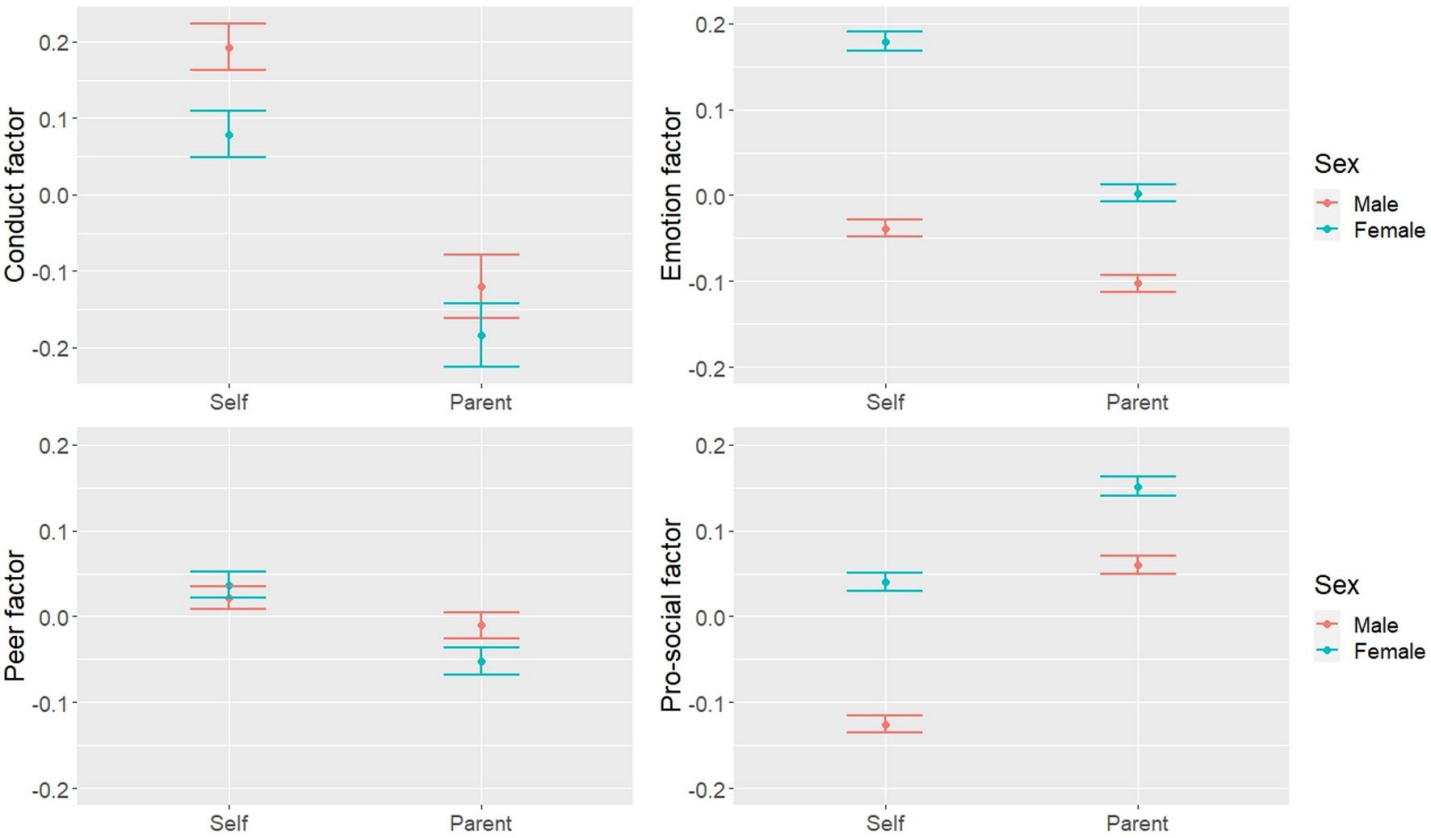
Predicted mean levels of SDQ factors by reporter and adolescent sex (95% confidence intervals)

LDS models conditional on adolescent sex (male/female) were tested by including sex as a predictor of both the self-reported and the discrepancy factors (Table 4). Sex was associated with three of the self-reported factors, as males reported higher conduct problems than females, and females reported higher emotion symptoms and pro-social behaviour than males. Sex was associated with all of the discrepancy factors, most strongly with emotion symptoms. The parameter estimates by group showed that while parents reported lower difficulties than adolescents overall, the discrepancy was greater among female adolescents for emotion and peer problems, and slightly greater among males for conduct problems. Parents reported higher pro-social behaviour than adolescents overall, but the discrepancy was greater in male adolescents (Fig. 2).

**Table 4:** LDS models conditional on adolescent’s sex

	Main effects on adolescent sex:		Parameter estimates by group:	
	Self-reported factor	Discrepancy factor	Males	Females
Conduct	− 0.369***	0.158***	− 0.977***	− 0.819***
Emotion	0.763***	− 0.397***	− 0.226***	− 0.623***
Peer	0.048	− 0.187***	− 0.103***	− 0.196***
Pro-social	0.647***	− 0.255***	0.632***	0.377***

Maximum likelihood estimation with survey weights; Female was the reference category; Standardised estimates shown for ease of interpretation; *p < .05, **p < .01, ***p < .001

#### Parental psychological distress

**Fig. 3:**
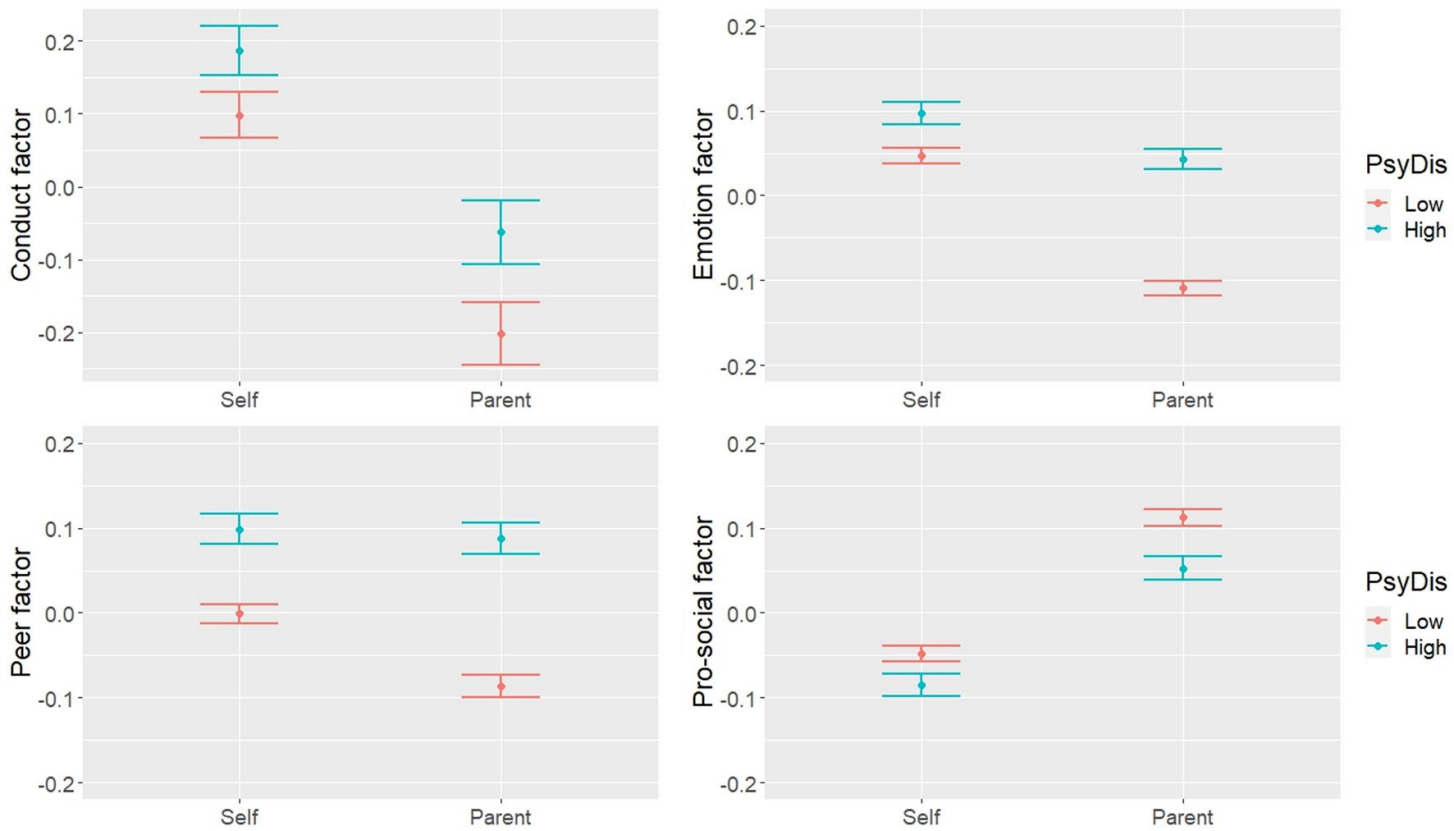
Predicted mean levels of SDQ factors by reporter and level of parent psychological distress (95% confidence intervals)

LDS models conditional on parental level of psychological distress (low/high) were then tested (Table 5). Parental psychological distress was associated with all of the self-reported factors, as adolescents reported higher difficulties and lower pro-social behaviour if their parent had a higher level of distress. Parental distress was associated with all of the discrepancy factors. The parameter estimates by group showed that parents reported lower difficulties than adolescents overall, but the discrepancy was greater if parents had a lower level of psychological distress, with the largest discrepancy observed for emotion symptoms. The same effect, albeit smaller, was observed for pro-social behaviour, as parents reported higher pro-social behaviour than adolescents, but the discrepancy was greater if parents had a lower level of psychological distress (Fig. 3).

**Table 5:** LDS models conditional on parent’s psychological distress

	Main effects on parent psych distress		Parameter estimates by group	
	Self-reported factor	Discrepancy factor	Low distress	High distress
Conduct	0.287***	0.161***	− 0.945***	− 0.784***
Emotion	0.178***	0.363***	− 0.553***	− 0.191***
Peer	0.308***	0.245***	− 0.281***	− 0.036
Pro-social	− 0.142***	− 0.067*	0.533***	0.457***

Maximum likelihood estimation with survey weights; Low distress was the reference category: standardised estimates shown for ease of interpretation; *p < .05, **p < .01, ***p < .001

#### Parental level of education

**Fig. 4:**
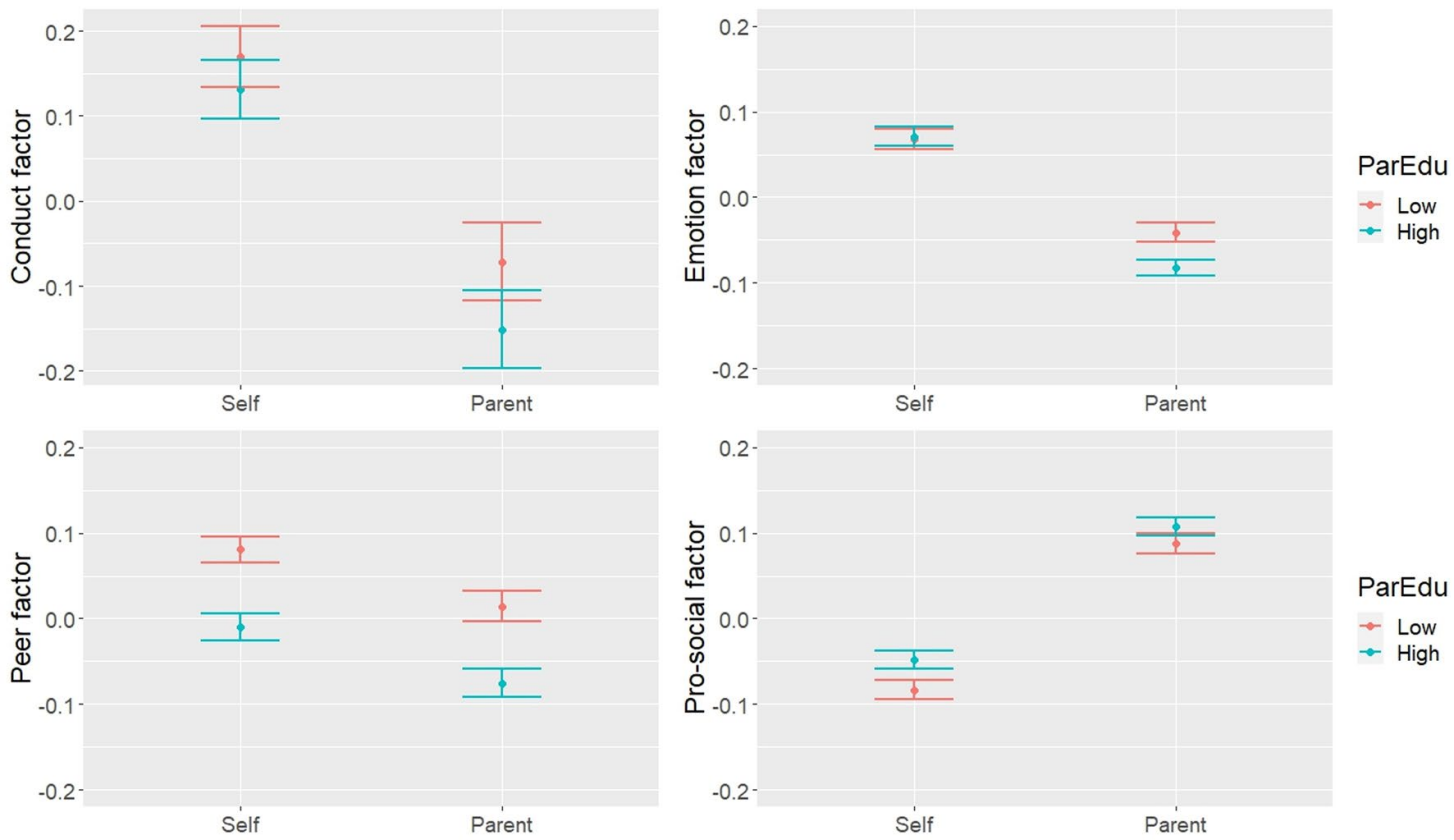
Predicted mean levels of SDQ factors by reporter and level of parent education (95% confidence intervals)

Finally, LDS models conditional on parental level of education (low/high) were tested (Table 6). Parental education was associated with three of the self-reported factors, as adolescents reported lower conduct and peer problems, and higher pro-social behaviour if their parent had a higher level of education. Parental education was associated with two of the discrepancy factors, as conditional effects were observed for emotion and conduct problems. The parameter estimates by group showed that parents reported lower emotion and conduct problems than adolescents, and the discrepancy was greater if parents had a higher level of education (Fig. 4).

**Table 6:** LDS models conditional on parent’s level of education

	Main effects of parent education		Parameter estimates by group	
	Self-reported factor	Discrepancy factor	Low education	High education
Conduct	− 0.120***	− 0.123***	− 0.736***	− 0.859***
Emotion	0.012	− 0.165***	− 0.399***	− 0.564***
Peer	− 0.261***	0.005	− 0.201***	− 0.196***
Pro-social	0.132***	− 0.052	0.578***	0.525***

Maximum likelihood estimation with survey weights; Lower level of education was the reference category: standardised estimates shown for ease of interpretation; *p < .05, **p < .01, ***p < .001

### Sensitivity analysis

We ran sensitivity analyses applying full information maximum likelihood estimation with robust standard errors to all LDS models (Additional file 2), as this was not possible in combination with survey weights. Estimates varied slightly, but results remained the same across all unconditional and conditional models. However, there was one case where the discrepancy factor became non-significant in the unconditional model for emotion symptoms, although the point estimate remained very similar.

## Discussion

In this study, parent-adolescent informant discrepancy was investigated for the first time on the SDQ, in the largest nationally representative birth cohort study of adolescents in the UK. Informant discrepancies were estimated using LDS modelling, and it was found that parents reported lower levels of difficulties (emotion, peer, conduct), and higher levels of pro-social behaviour than adolescents, which was in line with previous research in large scale general population samples in Chile [12] and Japan [24]. Findings are discussed below in the order they were presented in the results.

We were unable to find support for the proposed five-factor structure of the SDQ, which is not an uncommon finding [3, 15, 32, 34]. Previous confirmatory factor analyses conducted on the SDQ using MCS data have supported the five-factor structure at ages 5, 7, 11, and 14, but not age 17 [32–34], calling into question the acceptability of the SDQ factor structure in older adolescents. Despite this, most research using the SDQ examines individual mean scores of the subscales, therefore the approach taken in this study to examine factors independently can be considered valid. However, model fit was poor for the hyperactivity-inattention scale, therefore this construct was not explored further, perhaps because this scale is not unidimensional in older adolescents.

Measurement invariance was tested prior to estimating LDS models. Most factors showed weak (metric) invariance, while strong (scalar) invariance was not observed in any model. This is not an uncommon finding, as scalar level invariance is very difficult to support [29], but can be approximated through partial invariance [21]. The fact that full scalar invariance was not supported is perhaps unsurprising given that the wording is slightly different for some items between the parent and self-report version of the SDQ. For example, item 16 of the parent version from the emotion subscale (“nervous or clingy in new situations”) could have different connotations to the self-report version (“I am nervous in new situations”), especially among older adolescents. Indeed, this was one of the items that had to be freed across reporters. Despite these limitations, partial scalar invariance was established for all four remaining factors, by allowing one or two items to be freely estimated, which is in line with recommendations from the literature [38, 48]. While we acknowledge that this is not the ideal scenario, it better reflects the measurement attributes of the scale, rather than assuming invariance (and equal measurement properties across items) that underlies typical comparisons on the raw sum or mean scores [30].

### Discrepancy findings

To our knowledge, this is the first study to estimate latent difference scores among parents and adolescents on the SDQ. The largest parent-adolescent discrepancy was observed for conduct problems (almost 1 standard deviation difference), despite that externalising problems are often said to be more easily observed than internalising problems [2]. Parents may have underreported conduct problems due to either social desirability [23], or perhaps unawareness of adolescent’s behaviour, especially as participants were aged 17, when independence from parents is likely to be greater than at younger ages.

Discrepancies were also observed for emotion symptoms and pro-social behaviour (about half a standard deviation), with a minor amount of discrepancy observed for peer problems. The fact that parents tend to report more positive and less negative outcomes than adolescents suggests that on average parents have a general positive bias about their children. In light of this, future research would benefit from including a third more impartial reporter, such as teachers or peers, which could shed light on this further. An age effect could have also played a part in our study, as adolescents may have underreported positive and overreported negative outcomes due a general negative self-referential bias, which is characteristic of older adolescents [43]. Therefore, future research would benefit from assessing parent-adolescent discrepancy across different stages of development.

### Conditional effects

Conditional effects were observed for adolescent sex, whereby parent-adolescent discrepancy was greater for emotion symptoms and peer problems in female adolescents, and greater for conduct problems and pro-social behaviour in male adolescents. The largest effect was observed for emotion symptoms, as discrepancy was more than twice as large in parent-daughter dyads compared to parent-son dyads. This finding suggests that the large sex differences observed in adolescent emotion symptoms between males and females [36, 37], could be partly overestimated using self-report measures. However, emotion symptoms reflect internalising problems that may be more difficult to observe, particularly for parents with lower awareness of the associated signs and symptoms. Therefore, parents may be more likely to under-report symptoms in females, due to the higher degree of prevalence and severity in female adolescents.

Large sex differences were also observed for pro-social behaviour, whereby parent-adolescent discrepancy was greater in male adolescents. This could be related to the fact that items used to measure pro-sociality (e.g., kindness, consideration) are more analogous with female identity, and therefore possibly underreported by male adolescents. A recent study in a general population school sample of younger adolescents (median age 11 years) in Switzerland examined teacher–child informant discrepancy on pro-social behaviour and found that teachers reported lower pro-sociality across sexes, but to a greater extent in males [32]. Although this effect was in the opposite direction, as adolescents reported lower pro-sociality than parents in our study, together, this suggests that informant discrepancies for pro-sociality are likely to be greater in males, perhaps due to a general bias within society that associates pro-social behaviour with female identity.

The effect of parental psychological distress on discrepancy was significant for all four factors, as compared to parents with low psychological distress, reports from parents with higher levels of distress were more consistent with their children’s reports. This was evident for reports of pro-social behaviour, as well as all difficulties, suggesting that parents with higher levels of psychological distress may be less biased and more accurate at identifying both strengths and difficulties. This is in contrast to early research that questioned parental reports of children’s behaviour and adjustment when parents showed mental health difficulties [4, 39]. Our findings support other research in this area, that parents with mental health difficulties show more accurate reporting of children’s behaviour [8], perhaps because these parents are more sensitive and attuned to signs of maladjustment. It is also possible that awareness of children’s difficulties could be a causal factor explaining higher levels of parental psychological distress, although the direction of effects could not be disentangled in this study.

Parental level of education was associated with two of the discrepancy factors, as parent-adolescent discrepancy for emotion and conduct problems was greater at higher levels of parental education. This is somewhat surprising, especially since higher levels of parental education are known to be protective of children’s mental health and adjustment [43, 46]. Indeed, we found that adolescents themselves reported fewer emotion and conduct problems if their parent had a higher level of education. Therefore, the greater discrepancy cannot be attributed to a higher intercept for these scores. This finding suggests that higher educated parents may have a larger positive bias about their children than parents with a lower level of education. However, these conditional effects were small and reflect a novel finding, therefore should be replicated in other samples before drawing firm conclusions.

### Strengths and limitations

To our knowledge, this is the first study to examine informant discrepancies on the SDQ using a latent difference score approach. This approach has a number of advantages over traditional methods, including the possibility to test (rather than assume) measurement invariance, and calculate a true difference score that is as free from measurement error as possible [14]. In addition, the large and nationally representative sample used enable conclusions to be generalised to the population. A limitation of the study was the missing data observed on the SDQ. Nevertheless, a substantial proportion of the sample were analysed, and survey weights were constructed specifically for this study to help restore sample representativeness. Some benefits of the SDQ include its wide breadth of domains, covering internalising, externalising, and pro-social behaviour. However, model fit for the five-factor structure was found to be poor, therefore future research may wish to use other scales with better psychometric properties. We would expect the findings regarding parent-adolescent informant discrepancy to be consistent across other measures of adolescent adjustment.

## Conclusion

In a large general population sample from the UK, using novel methodology, this study found that adolescents reported more negative and less positive outcomes on the SDQ compared to parents. Discrepancy between reporters was found to be conditional on various factors, including adolescent sex, and parental level of psychological distress and education, showing that specific informant biases are likely to impact measurement of adolescent adjustment at each reporter level. To build on this research, future work would benefit from including more reporters, such as peers and teachers to give a fuller picture, as well as investigating discrepancy across different stages of adolescence, and including alternative multi-informant measures.

## Supplementary Information

**Additional file 1:** Univariate confirmatory factors analyses by subscale and reporter.

**Additional file 2:** Latent difference score models estimated with full information maximum likelihood and robust standard errors.

## Data Availability

All seven waves of Millennium Cohort Study data are available to download from the UK Data Service: https://ukdataservice.ac.uk/. An example of the R analysis code is available on the Open Science Framework: https://osf.io/yg485/
