# Supplementary material for: Parent-adolescent informant discrepancy on the Strengths and Difficulties Questionnaire in the UK Millennium Cohort Study
Source: Child Adolesc Psychiatry Ment Health. 2023 May 11;17:57. doi: 10.1186/s13034-023-00605-y (PMC10173568; doi:10.1186/s13034-023-00605-y)
Supplement: Supplementary file 1 — Additional file 1: Univariate confirmatory factors analyses by subscale and reporter. [file 13034_2023_605_MOESM1_ESM.docx]

**Supplementary File 1**

**Table 1.** CFA model fit and internal consistencies for SDQ subscales by reporter.

|  | Parent report | | | | | Adolescent report | | | | |
| --- | --- | --- | --- | --- | --- | --- | --- | --- | --- | --- |
|  | *N* | α | CFI | RMSEA | SRMR | *N* | α | CFI | RMSEA | SRMR |
| Conduct | 6624 | 0.59 | .905 | .061 | .035 | 6772 | 0.56 | .979 | .032 | .016 |
| Hyperactivity | 6655 | 0.76 | .796 | .194 | .071 | 6776 | 0.73 | .855 | .160 | .053 |
| Emotion | 6622 | 0.76 | .962 | .076 | .027 | 6756 | 0.74 | .976 | .065 | .023 |
| Peer | 6599 | 0.61 | .971 | .044 | .020 | 6787 | 0.56 | .984 | .030 | .014 |
| Pro-social | 6650 | 0.72 | .990 | .033 | .013 | 6795 | 0.66 | .985 | .038 | .016 |

***Note:*** *α = Cronbach’s alpha; Maximum likelihood with robust standard errors; Model fit was poor for hyperactivity-inattention across reporters and not analysed in the current study.*
