# Supplementary material for: Parent-adolescent informant discrepancy on the Strengths and Difficulties Questionnaire in the UK Millennium Cohort Study
Source: Child Adolesc Psychiatry Ment Health. 2023 May 11;17:57. doi: 10.1186/s13034-023-00605-y (PMC10173568; doi:10.1186/s13034-023-00605-y)
Supplement: Supplementary file 2 — Additional file 2: Latent difference score models estimated with full information maximum likelihood and robust standard errors. [file 13034_2023_605_MOESM2_ESM.docx]

**Supplementary File 2**

Latent difference score models estimated with full information maximum likelihood and robust standard errors. Results remained the same as the main analyses reported in the manuscript. However, in the unconditional model (Table 1) for emotional symptoms, the latent difference score became non-significant, despite the point estimate remaining largely the same as the original analyses.

**Table 1.** Latent difference score models between parent and self-report.

|  |  | | Model fit | | | Latent difference score | | |
| --- | --- | --- | --- | --- | --- | --- | --- | --- |
|  | *N* | CFI | | RMSEA | SRMR | Mean std | Variance | Covariance std |
| Conduct | 6947 | .928 | | .038 | .033 | -.790*** | .109** | -.470** |
| Emotion | 6947 | .964 | | .048 | .034 | -.459 | .087 | -.582 |
| Peer | 6947 | .952 | | .035 | .031 | -.182*** | .111*** | -.376*** |
| Pro-social | 6947 | .958 | | .040 | .034 | .521*** | .072*** | -.494*** |

***Note****: Full information maximum likelihood estimation with robust standard errors; Standardised means and covariances displayed for ease of interpretation; * p < .05, **p < .01, *** p < .001 level.*

**Table 2.** LDS models conditional on adolescent sex.

|  | Main effects on adolescent sex: | | Parameter estimates by group: | |
| --- | --- | --- | --- | --- |
|  | Self-reported factor | Discrepancy factor | Males | Females |
| **Conduct** | -.294*** | .122*** | -.852*** | -.731*** |
| **Emotion** | .753*** | -.399*** | -.253*** | -.652*** |
| **Peer** | .079* | -.177*** | -.091** | -.268*** |
| **Pro-social** | .676*** | -.281*** | .665*** | .384*** |

***Note:*** *Full information maximum likelihood estimation with robust standard errors; Female was the reference category; Standardised estimates shown for ease of interpretation; * p < .05, ** p < .01, *** p < .001.*

**Table 3.** LDS models conditional on parental psychological distress.

|  | Main effects on parent psych distress: | | Parameter estimates by group: | |
| --- | --- | --- | --- | --- |
|  | Self-reported factor | Discrepancy factor | Low distress | High distress |
| **Conduct** | .241*** | .263*** | -.889*** | -.626*** |
| **Emotion** | .220*** | .340*** | -.580*** | -.239*** |
| **Peer** | .314*** | .233*** | -.265*** | -.032 |
| **Pro-social** | -.127*** | -.142*** | .569*** | .427*** |

***Note:*** *Full information maximum likelihood estimation with robust standard errors; Low distress was the reference category: Standardised estimates shown for ease of interpretation; * p < .05, ** p < .01, *** p < .001.*

**Table 4.** LDS models conditional on parental level of education.

|  | Main effects of parent education: | | Parameter estimates by group: | |
| --- | --- | --- | --- | --- |
|  | Self-reported factor | Discrepancy factor | Low education | High education |
| **Conduct** | -.142*** | -.135*** | -.710*** | -.845*** |
| **Emotion** | .010 | -.181*** | -.372*** | -.552*** |
| **Peer** | -.263*** | -.055 | -.158*** | -.213*** |
| **Pro-social** | .130*** | -.008 | .542*** | .534*** |

***Note:*** *Full information maximum likelihood estimation with robust standard errors; Lower level of education was the reference category: Standardised estimates shown for ease of interpretation; * p < .05, ** p < .01, *** p < .001.*
